# Heatwave Early Warning Systems and Adaptation Advice to Reduce Human Health Consequences of Heatwaves

**DOI:** 10.3390/ijerph8124623

**Published:** 2011-12-12

**Authors:** Dianne Lowe, Kristie L. Ebi, Bertil Forsberg

**Affiliations:** 1 Department of Public Health and Clinical Medicine, Occupational and Environmental Medicine, Umeå University, SE 901 87 Umeå, Sweden; Email: bertil.forsberg@envmed.umu.se; 2 Centre for Health Communication and Participation, Australian Institute for Primary Care and Ageing, La Trobe University, 3086 Plenty Road, Bundoora, VIC 3083, Australia; 3 Department of Medicine, Stanford University, 260 Panama Street, Stanford, CA 94305, USA; Email: krisebi@essllc.org

**Keywords:** heatwave, adaptation, early warning system, prevention

## Abstract

*Introduction*: With climate change, there has been an increase in the frequency, intensity and duration of heatwave events. In response to the devastating mortality and morbidity of recent heatwave events, many countries have introduced heatwave early warning systems (HEWS). HEWS are designed to reduce the avoidable human health consequences of heatwaves through timely notification of prevention measures to vulnerable populations. *Objective*: To identify the key characteristics of HEWS in European countries to help inform modification of current, and development of, new systems and plans. *Methods*: We searched the internet to identify HEWS policy or government documents for 33 European countries and requested information from relevant organizations. We translated the HEWS documents and extracted details on the trigger indicators, thresholds for action, notification strategies, message intermediaries, communication and dissemination strategies, prevention strategies recommended and specified target audiences. *Findings and Conclusions*: Twelve European countries have HEWS. Although there are many similarities among the HEWS, there also are differences in key characteristics that could inform improvements in heatwave early warning plans.

## 1. Introduction

With climate change, extreme weather events such as droughts, hurricanes and heatwaves are increasing in frequency and intensity [1,2,3,4]. Negative health effects of exposure to heatwaves can include cramps, fainting, heat exhaustion, heatstroke, dehydration, disease exacerbations, combined effect of medications on thermoregulation and ultimately mortality. Heat affected individuals can progress to fatalities within a short time of exposure to high temperatures [5]. The true rate of mortality associated with heatwaves is often greater than initially attributed, as heat related deaths are not always recorded as such (for example, some are attributed to heart attack, cardiovascular or respiratory disease) [6]. The increase in mortality during a heatwave is preventable [7] and effectively preventing this risk has been highlighted as a priority issue by the World Health Organisation (WHO) and Euroheat [8,9]. 

Increases in heatwave related morbidity and mortality are attributed to increases in the frequency, intensity and duration of hot days, increased numbers of ageing adults and increased urbanization [10]. The heat island effect (a product of geometry, design, density, materials used, latent heat and decreased evapotranspiration due to reduced vegetation [11]) is partly responsible for the higher heatwave mortality associated with urban centres [12]. Combined with climate change, the heat island effect makes urban environments more susceptible to heatwaves and episodes of high air pollution, and causes air to cool more slowly than in less densely built up areas [11]. This stored heat at night is particularly problematic as mortality rates are higher in vulnerable populations when the average minimum temperature exceeds a threshold [13]. Populations vulnerable to heatwaves include the elderly, socially isolated, chronically ill, homeless, mentally ill, individuals with cognitive disorders and those taking medications that affect cognition, thermoregulation or have photosensitive side effects [14,15]. While those vulnerable to heatwaves are usually fragile and marginalised populations, relatively young people also experience excess mortality. Yet, heatwaves aren’t typically perceived as a health problem. Other extreme weather events, where the impact is more visible, are considered much more devastating.

While mitigation measures can reduce the impact of the heat island effect (such as climate responsive design; greening or white roofs, facades or parking lots; increased vegetation and irrigating grass), and climatic changes (through emission reduction and clean energy, for example), these measures take time to implement and have an impact. In the interim, there is also an urgent need for measures to reduce immediate mortality and morbidity impacts during heatwaves [16]. 

Heatwave early warning systems with response plans are an approach to reducing the human health consequences of heatwaves. Early warning systems involve forecasting the heatwave event, predicting possible health outcomes, triggering effective and timely response plans targeting vulnerable populations, notification of heatwave events, communication of prevention responses and evaluation and revision of systems [17,18,19]. After several devastating heatwave events in Europe highlighted the need to develop plans to effectively cope with heatwaves, many countries implemented HEWS as a risk reduction strategy [14]. Other countries, such as, Finland and Sweden are now developing HEWS. 

We conducted a scoping review to identify and characterize HEWS in European countries, including details on the threshold for triggering the early warning system, the various components of action plans, the nature of the messages and the ways messages are communicated to stakeholders, the recipients of the early warning systems, with the aim of informing the design or implementation of new plans for countries lacking systems and the evaluation and revision of current HEWS.

## 2. Experimental Section

We searched for policy or government documents outlining heatwave action plans for 33 European countries (European Union member and candidate countries, plus Norway and Switzerland). Internet searches were identified as the most appropriate methods to identify HEWS as there are few scientific papers on warning systems. The extensive searches were performed from March to May 2011, using the Google search engine to search for “heat health”; “heatwave”, “severe heat event”, “extreme high temperatures”, “action plan”, “early warning system”, “heat watch warning”, “management strategy” “heat adaptation” and “public health heatwave alert”. These terms were translated (using Google translate) into the relevant languages for each of the European countries searched. Additionally, we searched for relevant information from organizational websites such as meteorological, public health and government departments. Where appropriate documents were identified, the terminology used within the document to identify heatwaves and associated measures were used to further search for more recent documents. We also sought contact details of potentially relevant individuals from meteorological, health or environment departments where the search did not identify a HEWS document and emailed requesting further information. We translated the identified HEWS documents and extracted details on the trigger indicators (such as temperature, heat stress index or other measures), thresholds for action, notification strategies, mode of communication of risk, message intermediaries, communication and dissemination strategies, prevention strategies recommended and specified target audiences. We also contacted relevant individuals involved with the action plans to request that they validate the extracted data. The responses received were incorporated within the tables and the individuals providing responses are outlined in the acknowledgments.

## 3. Results and Discussion

### 3.1. Characteristics of Identified HEWS

We identified formal documented HEWS for 12 European countries. The documents typically outline a response at a national or regional level, but city specific responses were also identified for the Former Yugoslav Republic of Macedonia [20], Netherlands [21], and Italy [22]. A number of health department websites have heat advice documents, and a number of meteorological departments issue heatwave warnings. However, unless there is an integration of the two, and a document identifying at least one form of intervention and the steps taken following the trigger point for a heatwave, we didn’t consider these for inclusion in this review. The majority of the HEWS were either developed by the National Ministry of Health, or Environmental Ministry, or collaboration between these and other institutions such as meteorological services or a voluntary organization such as the Red Cross (e.g., Netherlands [21] heat action plan). See Table 1.

The HEWS use various indicators to trigger a heatwave (see Table 1). These measures include maximum temperature, heat index (which combines air temperature and relative humidity), synoptic (airmass) or other combinations of temperature and persistence. There are differences among plans in how thresholds are determined, with evidence that determining thresholds based on temperature-mortality relationships are more effective than basing thresholds on statistical cut-off points [23]. There is variability in what threshold would trigger an action plan and how much lead time organizations and the “at risk populations” are given to prepare. In a number of HEWS, the forecasted threshold must be exceeded on a number of days to trigger a warning. Typically national meteorological departments forecast or monitor the trigger indicator and notify the appropriate coordinating body, the health or environment minister or chief medical officer, when the trigger is met. See Table 1.

Some action plans considered other indicators and thresholds to either trigger an alert or upscale a level of alert or response in addition to temperature. Belgium [24], Hungary [25], Portugal [26], and Switzerland [27] include consideration of air pollution. This is primarily ozone and the responses are in line with a European Union Directive that information is to be released when ozone levels are 180 micrograms (one-millionth of a gram) per cubic meter air (µg/m^3^) and that the public is to be alerted when 240 µg/m^3^ are reached [28]. Health impacts increase as ozone levels increase. The UK [29] and Macedonian [20] plans mention a range of air pollutants and sources of further relevant information. A number of countries monitor mortality [20,22,25,26,27,29,30,31,32]. Typically mortality monitoring occurs in real-time or weekly and the threshold is a measure to upscale a level of response, rather than to activate the action plan. Other factors monitored within the HEWS are emergency service usage [26,30], drought, power failure [21], Ultraviolet (UV) radiation levels, fire or other local events [26]. See Table 2 below.

Heatwave action plans are typically organized into phases concerning “forecasting”, “monitoring”, “warning”, and “alert”. The levels of alert and their associated actions are often graded. Each heat action plan has its own terminology for the phases, (see Table 3) and series of associated actions.

### 3.2. Triggers, Thresholds and Notification

Reflecting differences in country climates and the acclimation of the populations [33], the thresholds and durations required to trigger an alert differ within the HEWS. For example, forecasted temperatures triggering a heatwave warning ranged from 27 to 32 °C (as a daily mean over a period of days). Within some plans, thresholds are outlined for particular regions or cities that reflect differences in acclimatization, the presence of urban heat islands, coastal experiences of heatwaves *versus* inland, and other issues. 

**Table 1:** European countries with identified heatwave early warning systems, temperature indicators and trigger thresholds.

Country	Developed by	Level	Year of identified plan	Frequency of updates	Heatwave alert coordinating body	Trigger Indicator	Threshold trigger	Lead time	Indicator monitoring/forecasting body
Belgium [24] accessed on 20 March 2011	Federal Public Service Health, Food Chain Safety and Environment	National	2008	Not described (updated 2011)	Unclear	Tmax; Tmin	3 day mean Tmax: ≥30 °C; Tmin: ≥18 °C	3 days	Uccule
France [30] Accessed on 11 March 2011	Ministry of Health and Sports	National and Regional plans	2010	Updated every year since 2004 (updated 2011)	Institut de veille sanitaire and Meteo-France	Tmax; Tmin HSI	3 days mean of Tmax > regionally dependent thresholds and 3 days mean of Tmin > regionally dependent thresholds, HSI: risk: 27 °C; high risk: 32 °C, danger: 41 °C	5 day forecast; 24 hr alert (NB: if >2 days of alert forecasted—bulletin also includes health indices)	Météo-France
Germany [34] accessed on 11 March 2011	Federal Environment Agency & German Weather Service	National with warnings on county level	2008	Initiated in 2004, update frequency not described	German Weather Service	PT, Tmin	Severe heat stress: PT ≥ 32 °C (exact threshold depends on weather situation of last 30 days but does not exceed 34 °C); Extreme heat stress: PT ≥ 38 °C. Warnings if thresholds are exceeded for 2 consecutive days and Tmin (night between) > 16–18 °C)	48 hour	German weather service
Hungary [25] accessed on 11 March 2011	The National Public Health Central Hungary	National (Local temp and morality monitoring—Budapest)	2006	Not described	The National Public Health co-ordinating body–Alert issued by National Chief Medical Officer	Tmean	3 day Tmean > 26.6 °C (98% frequency)	10 day forecast (Euroheat) & 3 day (MS) Heatwave alert	Euroheat and Meteorological service (MS)
Italy [22] Accessed on 16 May 2011	Department of Civil Protection and the Ministry of Health	National and local (outlines temp thresholds for 27 Cities)	2009–2011	Updates every two years since 2004 (Last updated 2011)	National: The Lazio Region Department of Epidemiology. Regional: Local Civil Protection, Municipality, or Local Health Authority	Tapp max	3 days Tapp max thresholds (increasing monthly) range: 25.5 °C to 37.5 °C for 10–20% excess mortality & for >20% excess mortality range: 27.5 °C to 39.5 °C. Airmass models: Excess mortality in 65+ population (%): Dry Tropical (DT): 7% to 20% Moist Tropical plus (MT+): 15% to 46% Moist Tropical (MT): 4% to 8.6%	Up to 72 hours in advance in 27 cities	Meteorological Service of the Department of Civil Protection, Ministry of Health
The Former Yugoslav Republic of Macedonia [20] accessed on 29 April 2011	Ministry of health (National Commission for Climate Change and Health)	National with 6 regional thresholds	2011	Annually	Ministry of health (National Commission for heat-waves protection )	Tmax	Specifies Tmax specific monthly trigger threshold for each of the 4 phases for 13 cities in 6 regions from May to Sept	2 day forecast; 24 hr alert	Macedonian meteorology
Netherlands [21] accessed on 5 May 2011	Ministry of Health, RIVM, the Netherlands Red Cross, and ActiZ GGD Netherlands	National	2007	Not described	National Institute of Health (RIVM)	Tmax	5+ days above 27 °C	At least 24 hours	Meteorological Services
Portugal [26] accessed on 18 April 2011	The Directorate-General of Health and Ministry of Environmental and Occupational Health	National (with regional thresholds)	2010	Annually (updated 2011)		Tmax, Tmin, plus regional Tmax, ICARUS index, HSI, data considered	6 day Tmax >5 °C higher than Tmax mean of 1961–1990 period; Yellow alert: Icarus 0.01 to 0.99, or 1 day observed Tmax + 2 days forecasted Tmax May–Sept ≥ 32 °C and <35 °C; Tmax all regions; ≥35 °C to <38 °C July–Sept Alentejo only, or Tmin: May–Sept ≤24 °C & <26 °C (all regions) 2 days Tmin observed & > 2 days Tmin expected. Red alert is triggered if the indices > yellow alert.	Daily 3 day forecast	Monitored and co-ordinated by Operation group health (GOS) *:
Romania [31] accessed on 11 March 2011	Public Health Ministry and Meteo Romania	National	2008	Not described	Ministry of Public Health	Tmax	Alert: Tmax 35–38 °C; Maximum response: Tmax 35–40 °C	48 hours	National Institute of Meteorology and Hydrology
Spain [32] accessed on 11 March 2011	Ministry of health; Directorate general of public health	National (with thresholds for each province, #52) NB each region has a HEWS	2011	Frequency not described (First iteration 2004)	The Ministry of Health	Tmax; Tmin	Tmax and Tmin (simultaneously) >95% of the past series (sic, ‘historical series’) for the next 5 days Some exceptions for some regions (*i.e.*, coastal northern region)	5 days The level is assigned depending on the number of forecasted days exceeding the threshold Level 0: 0/5 Level 1: 1–2/5 Level 2: 3–4/5 Level 3: 5/5	Monitored and co-ordinated by Ministry of Health (General Directorate of Public Health) and Ministry of Environment (Spanish Agency of Meteorology and Climate Change Spanish Office). Other ministries and agencies are also involved in the national plan.
UK [29] Accessed on 11 March 2011	UK Department of Health and the Welsh Assembly.	National (with regional thresholds)	2010	Annually (updated 2011)	Department of Health	Tmin, Tmax	Level 2 alert: 60% chance of trigger; Temp ≥ 30 °C by day & 15 °C overnight for 2 days and night b/n (range of regional Temps specified). Level 3 alert: heatwave conditions met in 1 or more regions.	Level 2: 2–3 days Level 3: 1 day of threshold conditions met and next day forecast meets threshold	The Met Office
Switzerland [27] accessed: 15 March 2011	The Federal Office of Public Health and the Federal Office for the Environment	National (with links to 3 regional plans)	2007	Last updated 2007	The Federal Office of Public Health and the Federal Office for the Environment	HI	NOAA’s Heat Index threshold: 90 as a max daily value—which equals an air temperature of 32 °C at a humidity of 40%.	The next day	Meteo Swiss

* Operation group health (GOS) consists of Environmental Health Alert System and Appropriate Response group, Department of Epidemiology and Health Statistics, Health Service, the National Health Authority, National Institute for Medical Emergencies , Department of Epidemiology, Meteorology Institute, National Authority for Civil Protection HEWS: heatwave early warning systems; Tmax: temperature maximum; Tmin: temperature minimum; HSI: Heat stress index, (intensity & duration of heatwave, air humidity, air pollution); °C: Degrees Celsius; InVS : Institut de veille sanitaire; PT: perceived temperature; Tmean: mean temperature; Tapp max: maximum temperature apparent, (air mass, humidity); Icarus index: (predicts the effects of heat waves on mortality).

**Table 2:** Monitoring of other health indicators (air pollution, mortality, morbidity).

Country	Air pollution (actions)	Mortality (actions)	Other health indicators
Belgium [24]	✓ Ozone; info phase threshold = 180 µg/m^3^; alert = 240 µg/m^3^. sends press releases to press agencies	✗	✗
France [30]	✗	✓ Mortality data from the National Institute of Statistics and Studies (INSEE) collected and sent to Institut de veille sanitaire (InVS) daily, analyses weekly	✓ Institut de veille sanitaire monitors health care data from OSCOUR (Organization for the Coordinated Monitoring Emergencies) for heat related illnesses. Also difficulty with drinking water or electricity supply, hospitals being overwhelmed with patients, or drought.
Germany [34]	✗ (Mentions ozone and air-pollutants as an added risk factor during heatwaves)	✗	✗
Hungary [25]	✓ Air pollution-ozone; info phase threshold = 180 µg/m3; alert = 240 µg/m3.	✓ Real-time National monitoring of mortality from May to Sept	✓ Real-time National monitoring of emergency ambulance calls from May to Sept
Italy [22]	✗	✓ Real-time National monitoring of mortality in 34 cities, data collected by local Municipal Registry Offices sent to National Coordination Centre. Data includes: date and place of birth and death, gender, residence and the cause of the event (accidental\non-accidental))	✗
The Former Yugoslav Republic of Macedonia [20]	✓ Sulphur dioxide, nitrogen dioxide, ozone and carbon monoxide	✓ (Daily mortality in Skopje (capital); records grouped by age and sex and compared with meteorological data)	✓ Monitors # of calls to emergency in 2 cities (Skopje and Strumica) daily (records and groups symptoms and compares with meteorological data)
Netherlands [21]	✗	✗	✓ Alert if water scarcity or power failure, can be scaled up to a crisis.
Portugal [26]	✓ Ozone; info phase threshold = 180 µg/m3; alert = 240 µg/m3.	✓ Daily mortality data fed back to GOS	✓ Monitors daily demand for emergency services, hospital admissions, 24 Health service demand from 15 May to 30 Sept. Ultraviolet, fire and local events
Romania [31]	✗	✓ Implements uniform same day reporting of death due to heatwaves	✓ # people fallen in the street; #. first aid points; #. requests to SAJ; % increase in the number of requests from the previous day to SAJ; # intervention mobilized ambulances in the county; # necessary additional ambulances; Staff SAJ enough; If not, what staffing; # control actions carried out by ISS
Spain [32]	✗	✓ (Monitors daily funeral data from 10 municipals, records of age/sex/death & daily coroner death certificates from 4 hospitals in Barcelona)	✗
UK [29]	✓ Sulphur dioxide, nitrogen dioxide, ozone and carbon monoxide	✓	✓ HPA monitors the number of calls people make to NHS Direct and the number of visits made to a sample of GP practices. Emergency level reached if power or water shortages
Switzerland [27]	✓ Ozone; info phase threshold = 180 µg/m^3^; alert = 240 µg/m^3^.	✓ (Monitors mortality weekly)	✗

**Table 3:** Description of the levels or phases in the HEWS.

Country	HEWS active period	Phases	Actions prior to heatwave	Actions during heatwave or heatwave forecasted
Belgium [24]	15 May to 30 Sept	3 phases: 2 levels of monitoring; and heatwave action phase	Forecasting, monitoring; warning (press release bulletin) (2 levels);	Heatwave alert; suggest public cooling areas, provides phone hotline (general).
France [30]	1 June to 1 Oct	3 phases: **monitoring** (1/6 to 31/8); **warning and action** (mise en garde et d’actions MIGA): heatwave forecasted & **maximum mobilization**: heat wave with implications beyond the health field	Monitoring: Seasonal surveillance & monitoring (1/6 to 31/8) leaflet and poster (seniors, parents athletes, workers); pre-heatwave prevention/ management media campaign; heat-hotline	Warning & action, & maximum mobilization: Upscale hotline staff, tracking & supporting homeless (lockers, t-shirt, water, sun-cap, map of drinking fountains, emergency shelter venues), voluntary registry of vulnerable, monitors vulnerable (NGO, including Red Cross, visits); installation & maintenance of air-conditioned commonroom in residential care; additionally health departments & residential institutions have white & blue heat action plans PM decides when to cease **maximum mobilisation** & communicates this decision to relevant stakeholders & ministries
Germany [34]	Not described	3 phases: **forecasting: pre-warning: official warnings**	Forecasting, monitoring; phoneline up to week in advance; pre-warning information, heat illness prevention and recognition information campaign	Phone in line; heatwave information -based official warnings: update daily-internet: website 24 hours notice.
Hungary [25]	Not described	3 phases: **Alert (Gr1)**: 25 °C (15% mortality increase); **Preparedness (Gr2)**: 3 days forecast ≥25 ºC or 1 day Tmean 27 ºC (15% mortality increase); **Alarm (Gr3)**: 3 days Tmean 27 ºC (30% increase mortality)	Alert: National Institute of Environmental Health notifies National Ambulance Service, pre-season awareness campaign	Forecasted: info to health care system & public. During heatwave, provide portable water in public places, water roads & parks in evenings, monitor water supply and quality, planned disruption of electricity. Extraordinary measures: increase hospital beds, ambulance units, hospital staff, cool bodies at morgues, extend opening hours public air-conditioned places and pools, transport action, defer non-essential surgery.
Italy [22]	15 May to 15 Sept	3 phases, Level 1 attention: pre-warning conditions & low risk of mortality, Level 2 alarm: meteorological conditions associated with a high risk. Level 3 Emergency : 3 or more consecutive days of level 2	Level 1: flyers in centres for elderly and public places, local pharmacies health centres and GPs. Information through national/local help-lines and via the media. Identification of at-risk subgroups sent to GPs and Health/Social services	Diffusion of warnings via the media, Ministry of Health/Civil Protection websites. Active monitoring of vulnerable subgroups by GPs, social workers, volunteers (phone calls & home visits by GPs). Activation of Emergency protocols in care and retirement homes and in hospitals (postpone non emergency surgery, discharge planning, staff rotation restrictions, increased hospital beds).
The Former Yugoslav Republic of Macedonia [20]	1 May to 30 Sept	4 phases. Phase 0 (green) no danger; phase 1 (yellow) alert temp , phase 2 (orange) Heatwaves; Phase 3: (red) dangerous (catastrophic) temp:	Monitoring, information provided to retirement homes & GPs, installation or maintenance of public drinking fountains & springs, education to public. Phase 1: preventative measures media campaign, home visits to elderly, socially isolated and homeless (red cross); phone line.	Phase 2: supply food to elderly & at risk media alert, specific measure for health care preparedness, protection measures for occupationally heat exposed workers; including activating redistribution in residential settings to air-conditioned rooms, extra staff on hotline, Phase 3: Emergency, lead by National Crises Management Center
Netherlands [21]	1 June to 1 Sept	3 phases: Vigilance; Diligence: heightened monitoring 20% probability of temps for 5 or more days reaching 27ºC, Warning Phase-probability of a period of five days or more above 27ºC is 90%.	Monitoring, institution aware of need to plan for heatwaves.	Media alert, press release from the National Institute of Health and Meteorological Services; assess needs (e.g., shelter) of homeless; volunteers or GP monitor vulnerable.
Portugal [26]	15 May to 30 Sept	2 phases: surveillance and daily alert; 3 alert levels: green, amber and red. Green Alert: normal temperatures, Amber Alert: high temperatures; Red Alert: serious high temperatures.	Surveillance. Monitoring and forecasting, Green alert: general measures of heat health information.	Amber alert: Info to public, authorities, health sectors, and media. Increase capacity of health care services, upscale staff for telephone hotline. Red alert: info to public, authorities, health sectors & media. Activate local refuge shelters, monitor need for transportation to places of refuge; notify most vulnerable (by referral); increase capacity of health care services.
Romania [31]	Seasonally when Tmax < 35 °C between the hours 11:00–17:00	3 phases: Code green (<35 °C b/n 11:00–17:00); Code Yellow (35–38 °C b/n 11:00–17:00) specific measures of alert; Code Orange (35–40 °C between 11:00–17:00); Code Red (>40 °C b/n 11:00–17:00) maximum mobilization measures.	Green: no specific measures; seasonal activation; yellow monitoring & support; general advice to public health authorities & monitors hospital needs (water supply, ventilation, storage tanks); municipalities to prepare list of persons with social dependency.	Free phone-in line (10:00–17:00); daily information to health ministry, health authorities; monitors sanitary, water, food prep, med storage, microclimate compliance of public services, housing & jobs, outreach people with social dependence (nurses & health mediators). Emergency response: according to public health priorities identified; increase support to ambulance or emergency services; hospitals emergency cases only.
Spain [32]	1 June to 15 Sept	2 phases :Phase 0: 1 June to 15 Sept; Phase 1: 15 June to 31 Aug.	Information, site specific actions, lists of at risk (AR) individuals, checks with regional services.	Dissemination of temperature and mortality data; prevention information to AR patients (personal fan health advice leaflets); general hotline activated; AR social services, monitoring (phone call &/ visit) vulnerable (referral through social services); relocation of residential AR populations to air-conditioned environment; activation of emergency services.
UK [29]	1 June to 15 Sept	4 phases. Green: Summer vigilance Yellow: Alert & readiness. Amber: Heatwave, T-threshold for one or more regions reached for one day & next night, & the forecast next day > 90% confidence. Red: Emergency situation, severe prolonged heatwave.	Green: monitors, preparedness & long-term cool & energy efficient planning (urban housing, workplaces, transport systems & the built environment). Yellow: Disseminates forecasts, leaflet (via pharmacies, GPs, NHS, advice centres, HPA & national websites, hospitals, care homes), identifies & monitors at risk. Homes at risk from hot weather can request, home audit using the Housing Health and Safety Rating System.	Amber. Social and healthcare services target specific actions at high-risk groups. Red: Emergency measures; potential discontinuation of public or sporting events, closure of schools; provision of local cool centres, reduce urban heat & deteriorating air quality by minimising unnecessary transport and energy use. Implications for trains, staged preventative measures at 22 °C, extreme precautions at 36 °C; deploy hot weather notices & bottled water supply, as well as measures to prevent track buckling; implications for power supply.
Switzerland [27]	Not described	4 phases: Caution: 27–32 °C; HI: <32 °C. Extreme caution: 32–41 °C; Danger: 41–54 °C; Extreme danger: HI >54 °C. NB. HI:> 32 °C	Monitoring, forecasting, and dissemination of heat illness recognition and prevention information	Warning to GOS&A & population; no broader adaption strategies mentioned

The trigger thresholds for and timing of notification vary but usually are based on forecasted temperatures and therefore preceded heatwaves (see Table 1). Because adversely affected individuals can rapidly progress to fatality following the onset of extreme temperatures [35], timely notifications should precede heatwave events and not occur on the day the mortality-threshold temperature is exceeded [5]. However, frequent or inaccurate warnings do not contribute to the credibility of the system, so the specificity of the triggers is important. While not typically discussed in the HEWS, the predictive characteristics of the heatwave prediction models and how they were established are covered elsewhere [19].

There was little to no information contained in HEWS describing when heatwave alerts were to be downgraded after the initial trigger forecast. Montero *et al*. 2010 suggest that HEWS which trigger the highest alert levels only in the initial days of a heatwave insufficiently address the greatest increases in mortality which occurs in the latter days of a heatwave as a result of the accumulated effects of prolonged exposure to high ambient temperatures. As such they suggest health-care activities aimed at reducing population exposure to high ambient temperatures should extend beyond heatwaves and alerts should persist after the initial onset of a heatwave [23].

### 3.3. Tailoring Messages for Vulnerable Populations

Passive heat avoidance is not enough to combat excessive heat health effects [14,36]. This suggests a need to actively raise awareness of potential health impacts and advice on protecting against and recognising heat illnesses, including dehydration, heat stress and heat stroke. Some HEWS provide vulnerable populations with a timely warning of impending heatwave, and outline preventive strategies to minimise the negative effects of intense heat periods, see Table 4. It has been suggested that advice should be targeted towards, and explicitly identify, those who are most vulnerable to the adverse effects of heat (elderly, particularly women, and those with chronic lung diseases), as the message may be ignored if blanket warnings are issued [10]. Several HEWS provide brochures tailored to specific vulnerable group(s). The French action plan provides multiple information brochures, each tailored to a vulnerable population including elderly; athletes (through sporting groups and centres); carers of children (through schools/childcare centres and leaflets targeting parents); carers of adults (through nursing homes and leaflets targeting carers), and workers in addition to generalized heat health individual behavioural advice. Advice tailored to the audience is better received than general advice. 

The elderly and low-socio-economic populations are disproportionately illiterate, may have visual or auditory impairment or include populations for whom the local language is foreign, potentially increasing their vulnerability [37]. To address the communication needs of these populations, many HEWS use plain language advice and visual communication (such as symbols to accompany verbal or written warnings). Research suggests printed material that offers a combination of visual and textual information is more effective at communicating messages than texts or symbols alone [38]. In addition, many HEWS provide information in multiple languages, and the UK [29] action plan is available in large print, Braille and on audio tape.

**Table 4:** Communication, notification and prevention actions outlined in the HEWS.

Action	Proportion of action plans addressing action
**Identifies dissemination methods**	**12/12**
**Passive communication**	**11/12**
Media release	10/12
Leaflet	8/12
Website	8/12
Radio	3/12
Newspaper	4/12
Other *(commercials (1), media kit on intranet (1), posters (1), dedicated phone in line (1))*	3/12
**Active communication**	**3/12**
**Individual level-general**	**2/12**
SMS	0/12
Email	2/12
Phone call	1/12
In person	1/12
Other (newsletter sign up)	1/12
**Specifies sources of messages**	**2/12**
GP	2/12
Health centre nurse	0/12
Pharmacist	1/12
Other	1/12
**Identifies at risk populations**	**11/12**
Elderly	11/12
Chronically ill	11/12
Medication groups	11/12
Homeless	3/12
Obese/unfit	8/12
Cognitive	8/12
Outdoor workers	7/12
Physically active	6/12
Children	11/12
Disabled	8/12
Socio economic status	3/12
Ethnic minorities	0/12
Tourists	1/12
Isolated	3/12
Gender	1/12
Drug/alcohol dependency	4/12
Refined to most vulnerable	2/12
Other *(Ramadan (1); institutionalized (1); people with fever (3), pregnant women(1))*	4/12
**Individual adaptation advice**	**11/12**
Heat avoidance	11/12
Limit physical/outdoor activity	11/12
Wear loose light colour clothes	9/12
Hydration	11/12
Cool homes	11/12
Cool body	11/12
Spend time in aircon env	4/12
Help vulnerable individuals	9/12
Seek advice for health problems	6/12
Seek advice changing medications use	5/12
Medication storage	2/12
Food handling/preparation	7/12
Replenish electrolyte intake	4/12
Protect against sunburn	4/12
Know forecasted temp	4/12
Monitor room temp	3/12
Travel by night/cooler hours	2/12
Rational working hours	2/12
Other (mitigation pale/reflective paint, shading sun-facing aspects (2), turn off unused appliances (4))	4/12
**Outlines carer child adaptation advice**	**9/12**
**Outlines carer adult adaptation advice**	**9/12**
**Outlines any carer adaptation advice**	**10/12**
Identify at risk individuals	7/12
Increase monitoring of at risk individuals	8/12
Heat audit rooms	7/12
Consider staffing issues	3/12
Provide AC or common cool room	6/12
Install thermometers/monitor room temp	4/12
Freq. change of bed linen & storage	4/12
Appropriate food	7/12
Appropriate hydration	7/12
Structural/mitigation (shading, painting, greening of sun facing aspects (1), water roofs, grasses & vegetation at night (1), water sun facing walls (1))	4/12
Other (avoid excessive cooling (<28 °C) (1), avoid plastic continence pants/pads (2), weight loss is a measure of dehydration, weigh regularly (1), discuss adjustments of meds with GP before heatwave (1), adjust physiotherapy schedules to outside 11–16:00 (1), collaborate with family and residence (1)).	7/12
**Considers broader support measures**	**11/12**
Suggests attend public cooling area	8/12
Provides list of cooling areas	6/12
Monitors/supports public cooling area	4/12
Maintains list of vulnerable individuals	2/12
Monitors vulnerable individuals	8/12
Provides outreach to identified vulnerable	5/12
Addresses shelter/water needs of homeless	3/12
Seeks to identify transport needy	2/12
Provides support for transport needy	0/12
Provides heat health phone line	8/12
Includes evacuation plan	1/12
Discusses power outage	1/12
Other	0/12
**Explains heat adverse health effects**	**11/12**
Provides advice only	11/12
Provides emergency contact list	9/12
Provides primary prevention advice	12/12
Provides secondary prevention	12/12
**Mitigation strategies**	**3/12**
**Addresses pre-existing beliefs**	**0/12**
**Stakeholder briefing/training**	**5/12**

The dissemination of heat advice to vulnerable populations typically involved websites, pamphlet distribution or media campaigns (methods of communication were active in 3 of 12 HEWS and passive in 11 of 12 HEWS, see Table 4). Research suggests that as some of the heat vulnerable populations are isolated and dependent, presenting verbal information is more effective than simply providing written brochures or fact sheets [38]. General populations that have sufficient information and perceive a threat as endangering tend to be motivated to take action. However, vulnerable populations of low socio-economic status tend not to be persuaded by media to change behaviour [39]. These individuals may be reached through more effective communication modes and community based strategies [39]. As such, 5 out of 12 HEWS (see Table 4) action plans provide outreach and phone calls to ensure that notification of heatwave and heat illness prevention and recognition messages were received. 

Within the notification strategies, none of the HEWS present heat health risk information in a statistical format. As research, on the identification of particular risk factors and risk reduction measures, develops, so will the options for presenting the risks associated with heat waves. A review of statistical formats [40] suggests that people are more persuaded to adopt a health intervention when its effect is presented using relative risk reduction that represents a proportional reduction rather than in absolute terms that represents a simple difference. However, little is known about how risk presentations affect actual behaviour [40].

### 3.4. Implications of the Timing of Heatwaves

All but one HEWS is active between mid May and September and dormant throughout the rest of the year. However, the active HEWS period may need to be reassessed with time, as climate change may create less predictable seasonal effects. Heatwaves coincide during periods when institutions and people are in holiday mode. This has implications for the natural supports for vulnerable populations and the ability of emergency services and institutions to respond. The HEWS typically address potential negative implications by outlining plans to recruit, increase or recall staff to respond to heat related emergency situations (see Table 2); and outline strategies for the general public regarding planning for individuals in their care during the holidays. Other actions during a heatwave that were health care based include the capacity to postpone non-emergency services, and increase hospital beds and ambulances.

Even while acknowledging that typically heatwaves occur during periods when individuals take holidays, HEWS also focus on solidarity and the actions of neighbours, volunteers and carers by emphasizing the need to reach out to vulnerable populations because heat impacts are not just a medical or physical problem, but also a social problem. Socially based risk factors associated with mortality during heatwaves include being confined to bed, not leaving home daily and being unable to care for oneself [41]. Living alone during a heatwave was non-significantly associated with an increased risk of mortality, while participating in social activities was protective [41]. 

### 3.5. Heat Adaptation Advice

A number of novel social adaptation strategies to counter social risks are suggested in various HEWS documents. These included befriending, and outreaching to at-risk neighbours. It is also suggested that for carers of those at risk, notifying others (neighbours, parents, doctor or nurse on call) of planned holidays and finding someone to take over care. Other carer measures include preparing a hidden spare key, lists of medications, medical history and important numbers, and arranging to phone at predetermined times. In addition, vulnerable population outreach through voluntary services is also considered in some action plans (see Table 4). 

The majority of HEWS focus on improving heat health responses of residential staff and centres, through identification and increased monitoring of the most vulnerable, heat auditing rooms, daily monitoring of room temperatures and measures to cool environments and residents. As one plan stated “We often forget that a little human warmth (which does not influence the ambient temperature) helps older people to be more inclined to drink and eat regularly!” [24]

Other novel adaptation strategies target outdoor workers/physical active work through rational organization of work schedules (such as start work early, take breaks often, scheduling most physical activity early morning or late afternoon). Three HEWS consider support measures for the homeless, including shelters, provision of caps, lockers for storing belongings and maps of drinking fountains. One HEWS considers Ramadam, suggesting that the period of fasting from extra beverages and food during the day may have added heath implications during heatwaves [24]. 

Many action plans recommend spending two hours in an air-conditioned environment to reduce impacts of heat. This is in line with studies demonstrating air-conditioning use as a protective factor against mortality in heatwaves [41,42] and that ownership and usage of air-conditioners reduces the health effects of increases in same-day apparent temperature on risk of hospitalization for a number of diseases [43]. However, a study of middle-aged and older adults with chronic conditions living at home demonstrates that while perceived health sensitivity to heat was a significant predictor of using an air-conditioner, an extreme heat warning or advice from their doctor were not [44]. 

Increasing household air-conditioning use as a response to heat is problematic when the source of electricity contributes to greenhouse gas emissions; air-conditioning use also increases peak electricity demand and blackouts during heatwaves, which leaves at risk individuals more vulnerable to the effects of heat [45]. Encouragingly rather than recommending that people install air-conditioners (which many heat vulnerable individuals may not be able to afford to purchase or use), HEWS typically recommend provision of public places as a refuge during a heatwave and/or provision of an air-conditioned common-room for residential care environments. The refuges recommended are places where air-conditioning use cannot be avoided, such as in hospitals, department stores and public institutions. Typically these public places can justify a backup generator so are less affected by blackouts during heatwaves. In some HEWS, the hours of operation are extended during a heatwave and additionally, one HEWS lists heat-first aid centres within the listed refuges. 

Many plans place caveats around use of air-conditioning and fans for cooling. For air-conditioning, the caveat is that the temperature should be set to 5 degrees below the ambient temperature. There was little discussion surrounding the evidence of this advice. Fans can increase dehydration in rooms exceeding 35 degree Celsius temperatures. A number of HEWS also stress using tepid water to cool bodies to avoid the stress of sudden changes in body temperatures caused by cold showers/pool or drinking chilled water. A rather comprehensive review of effectiveness of personal heat health advice is presented elsewhere [46]. 

More than half of HEWS (see Table 4) contain advice on food hygiene, preparation and storage. In excessive temperatures, food spoils sooner, and rates of food poisoning can increase; food poisoning can increase dehydration and exacerbate the ill effects of heat. Related to food preparation, some action plans mention changing diet to include foods that are high in moisture content and served cool, and avoiding high calorie and hot foods that increase body temperatures. Avoiding cooking/heating foods also help keep buildings cooler. Cooking outdoors (such as BBQ) reduces heat inside; however, as drought and winds can accompany heatwaves, the use of fires outdoors is to be avoided during heatwaves. 

### 3.6. Heatwave Mitigation

Preventive strategies that doubled as mitigation strategies, such as structural changes to buildings to aid passive cooling and/or protect buildings against heating up, are often considered within HEWS. These included providing incentives for retrofitting wall and ceiling insulation; installation of awnings, blinds, insulated or double glazed glass, tinting windows (alternative temporary suggestion was foil covered cardboard placed in windows to reflect sunlight); planting trees to shade or white painting sun facing aspects; and replacing appliances and lighting with energy efficient and less heat producing forms and switching off unused appliances at power points particularly at night, which reduce strain on electricity supply and the heat emitted from appliances helping to keep houses cool. Other mitigation strategies discussed included long-term planning to decrease emissions by using renewable energy and energy efficient appliances; reconstruction, compensation or preservation of natural parks and environs (e.g., wetlands); greening (roof top, terrace and house gardens; tree planting in alleys, parks, groves, road side, meadows, and forests); carbon storage and fixing; restructuring traffic and transportation (active e.g., cycling/walking and public transport), and building zero emissions hospitals (mitigation-focus in Hungarian [25], the Former Yugoslav Republic of Macedonian [20] and UK [29] action plans). 

### 3.7. Comparison with Past Research

The Euroheat project, which was conducted from 2005 to 2007, undertook a similar assessment European HEWS [8,9]. While the Euroheat project involved individuals from within the European countries, the current project identified the HEWS through internet searches. At the time, Euroheat identified 16 HEWS. In contrast, we identified 12 HEWS, of which 10 overlap with those identified by Euroheat. The majority of the overlapping HEWS have been updated, (Spain, England, France, Hungary, Italy, Portugal Germany, Belgium, Switzerland and Netherlands) and 2 (Macedonia and Portugal) HEWS were introduced since the Euroheat survey was conducted. We did not include early warning systems for Slovenia, Slovakia and Lithuania, Israel, and Luxembourg identified within the Euroheat project as they were either not identified during the internet search or otherwise did not fit inclusion criteria (*i.e.*, we could not identify public health activities, dissemination or interventions associated with the heatwave warning). A direct comparison between the HEWS identified within the two projects is problematic due to differences in methods.

### 3.8. Limitations of This Study

The methods used in this scoping review of HEWS have some limitations. The first limitation is the methods used to identify the HEWS. As there is a lack of published data on HEWS for individual countries, we elected to use internet search to identify HEWS. A limitation of this method is that it may identify out of date documents, or in some instances completely missed some HEWS. Despite this limitation, we were able to identify two new HEWS, and a number of updated HEWS, which will aid in further informing the current state of HEWS development in Europe. 

The second limitation of this study is that the search for the HEWS documents took place from March to May 2011, which proceeded the summer period. Since the search was conducted a small number of HEWS have been updated (see Table 1). This means that there is the potential for information identified to be outdated. However given that efforts were made to contact individuals to validate the information extracted, the data is a fair representation of the state of HEWS at the time the research was undertaken. We were unable to validate the data for six countries, either due to lack of response from, or an inability to locate contact details for, appropriate individuals with knowledge of the country’s HEWS. A study based on a questionnaire (or request for information only) would have likely not identified or included these systems.

The third limitation of this study is that this scoping review is not an evaluation of HEWS, as there is no information available on the comparative effectiveness of the individual HEWS contained within the documents included. This is an area that urgently requires further research.

## 4. Conclusions

Twelve European countries have HEWS. While there were differences in the heat action plans, there are also many commonalities between the plans, such as involvement of meteorological institutions, types of indicators, actions, and vulnerable groups identified. The main features typical of a HEWS are timely accurate warnings, tailored communications and notifications of adaption actions to the most vulnerable populations and heat avoidance advice to general populations. Unfortunately, evaluations of the effectiveness of predicting heatwaves, notifying vulnerable populations, and adoption of adaption advice associated with communications are not currently available, and are urgently required to inform good practices. Current action plans may wish to incorporate some of the less frequently observed actions, such as monitoring the need for and providing transport to those as most risk, addressing the shelter and hydration needs of the homeless, maintaining a list of vulnerable individuals and using active individualised communication modes such as emails, SMS, phone calls to notify them of need to activate their personal heatwave adaptation measures. However, we acknowledge it is not appropriate for all heat action plans to be the same, because some regions experience unique climate effects and some populations are acclimatized to particular environments and the resources available will determine the actions taken or offered within each HEWS. 

Better understanding particular measures to increase resilience to heatwaves can improve existing and enhance current action plans. Future research is needed to address what actions were actually taken during HEWS compared to those recommended as well as evaluations of the effectiveness of the actions. It would be helpful for others planning on developing a heatwave early warning system to understand the process by which a system was established, how choices were made on such issues as triggers, how communication materials were developed and tested, what issues arose during implementation that could be prevented, institutional arrangements and how the system was designed for future evaluation. It would be more efficient for lessons learnt to be communicated than to be re-learned.
